# Concurrent appendiceal and umbilical endometriosis: a case report and review of the literature

**DOI:** 10.1186/1752-1947-8-258

**Published:** 2014-07-22

**Authors:** Daniel Paramythiotis, George Stavrou, Stavros Panidis, Dimitris Panagiotou, Kyriakos Chatzopoulos, Vasileios N Papadopoulos, Antonios Michalopoulos

**Affiliations:** 1 First Propedeutic Department of Surgery, AHEPA University Hospital, Aristotle University of Thessaloniki, St Kyriakidi 1, 54636 Thessaloniki, Greece; 2 Department of Pathology, Aristotle University of Thessaloniki, St Kyriakidi 1, 54636 Thessaloniki, Greece

**Keywords:** Endometriosis, Appendiceal endometriosis, Umbilical endometriosis, Concurrent endometriosis, Multifocal endometriosis

## Abstract

**Introduction:**

Endometriosis affects 3 to 10 percent of women of reproductive age. Most of the time it involves the pelvis; however, sites of endometriosis have been reported almost anywhere in the body. Appendiceal and primary umbilical endometriosis are considered rare loci, making accurate diagnosis elusive. Here we present the case of a 46-year-old woman with concurrent appendiceal and umbilical endometriosis.

**Case presentation:**

A 46-year-old Greek woman presented with a large mass in the lower abdomen adhering to the surrounding organs. She reported recurrent lower abdominal and pelvic pain and the presence of a dark-blue hard nodule at the umbilicus. She had no previous medical, surgical or gynecological history. Her physical examination and laboratory test results were without any significant findings. The laparotomy revealed a fibromatose uterus adhering to the rectum and a urinary cyst and a palpable mass in the vermiform appendix. A hysterectomy and an appendectomy were performed. The umbilical mass was also excised. Pathology revealed endometriosis of the umbilicus and the appendix. The postoperative period was uneventful and she was discharged.

**Conclusions:**

Endometriosis, although rare, should always be considered in women of reproductive age, presenting with cyclic pain. The diagnosis is, most of the time, difficult and requires a high degree of clinical suspicion. The clinical doctor should be aware that endometriosis can sometimes be multifocal, thus a thorough investigation is required in all cases.

## Introduction

Endometriosis has been defined as the growth of functional endometrial tissue outside the uterine cavity [1]. It affects 3 to 10 percent of women of reproductive age, presenting with symptoms such as dysmenorrhea, noncyclic pelvic pain, infertility, or menorrhagia [2]. Endometriosis usually affects the pelvis; however, extrapelvic involvement is not rare [3].

Umbilical endometriosis is quite rare, with a reported incidence of 0.5 to 1 percent, usually affecting patients after laparoscopy or other surgical procedure involving the umbilicus. Primary umbilical endometriosis is even rarer and the pathophysiology of this condition is not totally clarified [4]. Furthermore, appendiceal endometriosis presents in 0.8 percent of patients, with symptoms ranging from unclear abdominal complaints to those of an acute appendicitis [5].

We present a case of a synchronous appendiceal and umbilical endometriosis in a 46-year-old patient, as well as a review of the literature.

## Case presentation

**Figure 1:**
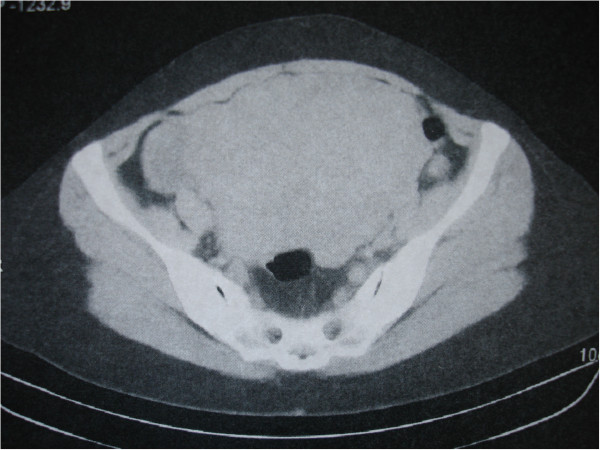
**Computed tomography image.** Large mass occupying the lower abdomen.

**Figure 2:**
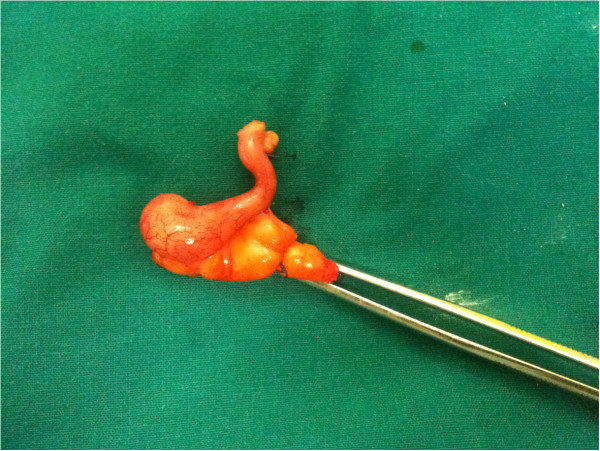
**Postoperative specimen of the vermiform appendix.** The mass is visible at the tip of the appendix.

**Figure 3:**
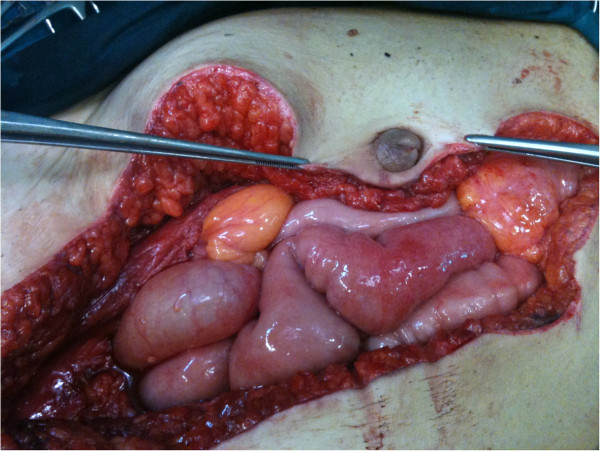
Intraoperative image of the umbilical lesion.

**Figure 4:**
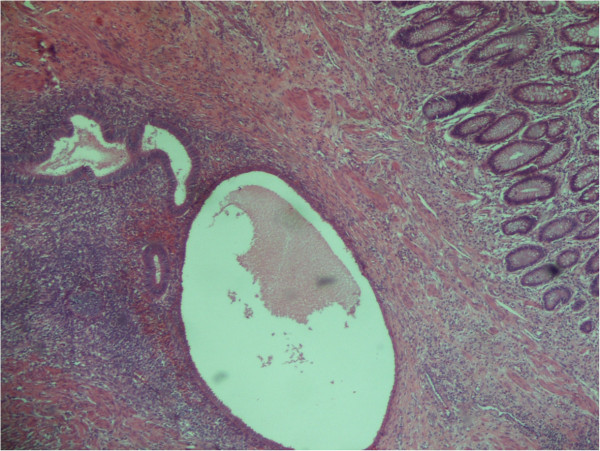
**Appendix (×40).** Endometrial foci in submucosa and muscular layer.

**Figure 5:**
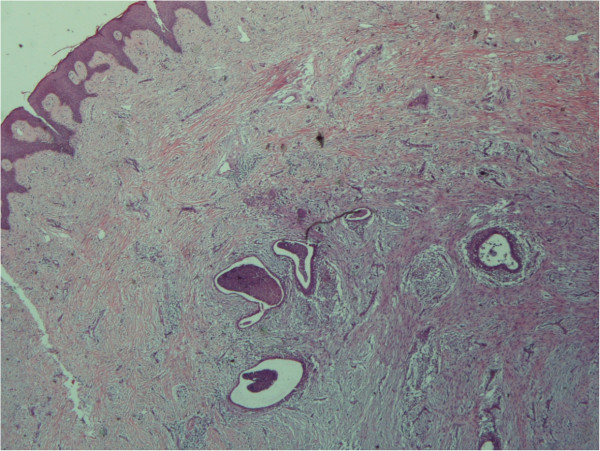
**Skin (umbilicus) (×20).** Endometrial foci in dermis.

A 46-year-old, Greek woman was admitted to our surgical department under investigation for a large pelvic tumor involving the uterus, rectum and urinary bladder, discovered previously during a computed tomography (CT) scan (Figure 1). She reported recurrent lower abdominal and pelvic pain and the presence of a dark-blue hard nodule at the umbilicus. She had two prior vaginal deliveries, and no previous medical, surgical or gynecological history.A physical examination was without significant findings. Her laboratory test results, as well as tumor markers (including cancer antigen 125 (CA-125)), were also within normal range. A colonoscopy was also without pathological findings. A laparotomy was performed and a large fibromatose uterus was discovered (size 19.5×12.5×12cm), with hard adhesions to the rectum and urinary bladder. On exploration of the abdominal cavity, a small tumor was palpated at the tip of the appendix (Figure 2). A total hysterectomy and a typical appendectomy were performed. The umbilical nodule (Figure 3) was excised and the umbilicus was reconstructed.Pathology revealed the presence of multiple large leiomyomas of the uterus, with a diameter ranging from 1.2 to 8cm. The appendiceal tumor (Figure 4) and the umbilical lesion (Figure 5) were found to be sites of endometriosis. Signs of malignancy were not detected in any of the three specimens.

The postoperative course was uneventful and she was discharged on the fifth postoperative day. At her six-month follow-up, she remained asymptomatic and in good condition.

## Discussion

Endometriosis concerns a chronic disease defined as the growth of functional endometrial tissue in sites outside the uterine cavity. The commonest sites of endometriosis involve the pelvis; the ovaries, the ureterosacral ligaments and Douglas pouch [6,7]. However, extrapelvic development of endometriosis is not rare, since ectopic endometrial tissue has been observed in the gastrointestinal tract, urinary system, liver, diaphragm, pleura, lung, brain, cutaneous tissue, pericardium, eye and other sites [3]. There are also reports of endometrial foci development in men, most often associated with estrogen therapy for prostate cancer; to the best of our knowledge, there have not been any reports of endometrial tissue development in the spleen [8]. Furthermore, it should be mentioned that endometrial lesions, especially those located in the colon, have the potential of malignant alteration [9].

Various theories have developed in an attempt to explain the pathogenesis of endometriosis. The retrograde menstruation or implantation theory, first proposed by Samson in 1927 [10], suggests that during menstruation endometrial tissue refluxes through the fallopian tubes and onto the nearby organs. Halban [10] proposed a direct transportation of endometrial cells via blood or lymph vessels or even through surgical manipulations. The embryonic rest theory suggests that a specific stimulus to a Müllerian origin cell nest produces endometrial foci [11], while the coelomic metaplasia or induction theory proposes metaplasia of peritoneal mesothelial tissue cells into endometrial cells, a situation induced by substances secreted from the shed endometrium, hormonal manipulations or chronic inflammation [12]. Finally, other theories suggest that alterations in the cellular and humoral immunity response promote development of ectopic endometrial cells [13], while under careful examination is the role of stem/progenitor endometrial cells [14].

Endometriosis of the appendix accounts for 0.8 percent of patients with endometriosis. Appendiceal endometriosis can manifest with a variety of symptoms, ranging from asymptomatic to a ‘typical’ acute appendicitis. Furthermore, appendiceal endometriosis can present serious gastrointestinal complications, such as intussusception, melena, lower gastrointestinal bleeding or even bowel perforation [15]. Appendectomy is the treatment of choice. Laparoscopic appendectomy allows a thorough exploration of the entire abdominal cavity, especially in patients with unclear or recurrent abdominal complaints.

Umbilical endometriosis was first described by Villar in 1886, and has been described as Villar’s nodule ever since [10]. Its incidence is reported to range from 0.5 to 1 percent in cases with endometriosis [16]. It is usually associated with laparoscopy, umbilical hernia surgery or any other intervention involving the umbilicus. Primary umbilical endometriosis is an even rarer condition, and its pathophysiology has not yet been totally clarified [4]. It usually presents with a dark nodule at the umbilicus. Its size may vary, following the menstrual cycle, and is often accompanied by cyclic pain [16]. However, there are many atypical cases, so the differential diagnosis between endometriosis and other soft tissue tumors can be quite difficult. In published series, umbilical endometriosis is misdiagnosed in 20 to 50 percent of the cases [4].

The treatment of choice in all cases of abdominal wall endometriosis is a wide resection of the lesion, if necessary with partial resection of the underlying fascia. For most lesions, a margin of 1cm is considered adequate [2,10]. If the umbilicus cannot be preserved, it should be reconstructed using various plastic surgery techniques, while postoperative abdominal wall defects may require the use of a mesh.

After surgical treatment, a thorough gynecologic assessment is strongly advised to fully determine the extent of the disease [5], and when appropriate, to prescribe additional hormonal treatment (that is, gonadotropin-releasing hormone (GnRH) analogs, progestagens, oral contraceptives, levonorgestrel intrauterine system or less often danazol).

## Conclusions

Endometriosis has been proven to be a possible cause in cases of unclear/atypical abdominal pain, especially in women of reproductive age. Clinical manifestations are often puzzling and misguiding, so a surgical exploration, by means of laparoscopy/laparotomy is often necessary for the final diagnosis. In cases where cutaneous development of endometrial foci is discovered, a thorough clinical and radiologic evaluation is advised, to exclude any possibility of multifocal disease.

## Consent

Written informed consent was obtained from the patient for publication of this case report and any accompanying images. A copy of the written consent is available for review by the Editor-in-Chief of this journal.

## Abbreviations

CA-125: cancer antigen 125; CT: computed tomography; GnRH: gonadotropin-releasing hormone.

## Competing interests

The authors declare that they have no competing interests.

## Authors’ contributions

DP, VNP and AM performed the operation and critically revised the manuscript. KC performed the histological evaluation and provided a valuable contribution to the drafting of the manuscript. GS, SP and DP collected the data and drafted the manuscript. SP critically revised it. All authors read and approved the final manuscript.
